# A naturally occurring omega current in a Kv3 family potassium channel from a platyhelminth

**DOI:** 10.1186/1471-2202-9-52

**Published:** 2008-06-19

**Authors:** Tara L Klassen, Andrew N Spencer, Warren J Gallin

**Affiliations:** 1 Department of Biological Sciences, University of Alberta, Edmonton, Alberta, T6J 2E9, Canada; 2 Malaspina University College, 900 Fifth Street, Nanaimo, British Columbia, V9R 5S5, Canada

## Abstract

**Background:**

Voltage-gated ion channels are membrane proteins containing a selective pore that allows permeable ions to transit the membrane in response to a change in the transmembrane voltage. The typical selectivity filter in potassium channels is formed by a tetrameric arrangement of the carbonyl groups of the conserved amino-acid sequence Gly-Tyr-Gly. This canonical pore is opened or closed by conformational changes that originate in the voltage sensor (S4), a transmembrane helix with a series of positively charged amino acids. This sensor moves through a gating pore formed by elements of the S1, S2 and S3 helices, across the plane of the membrane, without allowing ions to pass through the membrane at that site. Recently, synthetic mutagenesis studies in the *Drosophila melanogaster *Shaker channel and analysis of human disease-causing mutations in sodium channels have identified amino acid residues that are integral parts of the gating-pore; when these residues are mutated the proteins allow a non-specific cation current, known as the omega current, to pass through the gating-pore with relatively low selectivity.

**Results:**

The *N.at-K*_*v*_*3.2 *potassium channel has an unusual weak inward rectifier phenotype. Several mutations of two amino acids in the voltage sensing (S4) transmembrane helix change the phenotype to a typical delayed rectifier. The inward rectifier channels (wild-type and mutant) are sensitive to 4-aminopyridine (4-AP) but not tetra-ethyl ammonium (TEA), whereas the delayed rectifier mutants are sensitive to TEA but not 4-AP. The inward rectifier channels also manifest low cation selectivity. The relative selectivity for different cations is sensitive to specific mutations in the S4 helix,

**Conclusion:**

*N.at-K*_*v*_*3.2*, a naturally occurring potassium channel of the Kv3 sequence family, mediates ion permeation through a modified gating pore, not the canonical, highly selective pore typical of potassium channels. This channel has evolved to yield qualitatively different ion permeability when compared to all other members of this gene family.

## Background

When voltage-gated potassium channels are activated, the S4 voltage sensor helices translocate and rotate a short distance within a gating-pore [1-4]. This restricted conformational change moves a number of charges across the membrane electrical potential field. Movement of S4 segments may be as little as 4 Å, and is augmented by the electric field being focused and swept across some of the N-terminal charges on each S4 segment as the voltage sensor moves [2,5]. The gating pore includes an aqueous canal, contiguous with the intracellular solution located at the interface of the pore and voltage-sensing domains [4,6-8], that acts to focus the local electric field within the membrane across a small portion of the S4 voltage sensor [9]. A small, ~3Å, hydrophobic septum that acts as a barrier to ion current through the gating pore is maintained by interactions between S4 and the rest of the voltage sensing domain [2,10-13].

The N-terminal arginine (R1) in the S4 segment of the *D. melanogaster *Shaker channel sterically hinders ion passage through the gating-pore [12]. Substitution of the first arginine (R1) in the voltage sensor with histidine, R362H, allows a H+ current to flow at hyperpolarized, subthreshold potentials [2], while anR371H mutant allows a proton flux at depolarized potentials [14]. Mutations of the R1 (R362) position in Shaker to alanine, cysteine, serine, and valine allow influx of a non-selective cation current, the "omega current", through the gating pore [12,15]. Recently a gating-pore mutation in the sodium channel Nav1.4, has been implicated as the molecular basis of hypokaelemic periodic paralysis [16]. In this channel, mutation of residues R2 or R3 in one of the four S4 helices allows an influx of cations through the aqueous gating-pore along the length of S4. The omega current in both cases is present only when the channel is in a hyperpolarized conformation. When the channel is in a depolarized conformation, the omega pore closes and the canonical ion-selective pore opens.

**Figure 1:**
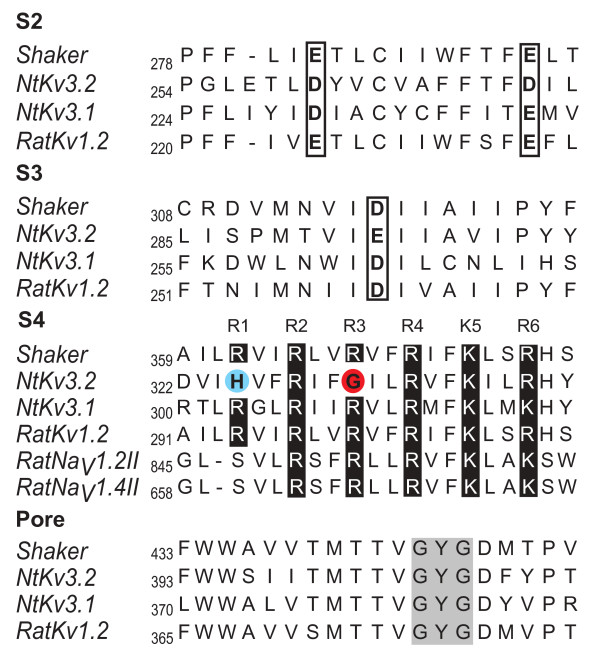
**Distinctive sequence characteristics of *N.at-K*_*v*_*3.2 *from *Notoplana atomata***. Amino acid sequence alignment of the S2, S3, the S4 voltage sensor and the canonical pore of *N.at-K*_*v*_*3.2 *with other voltage-gated ion channels (*Shaker – Drosophila Shaker *B; *Nt Kv3.2 – Notoplana atomata Kv3.2 channel; Nt Kv3.1 – Notoplana atomata Kv3.1 channel*;*RatKv1.2 – Rattus norvegicus Kv1.2 channel;RatKv1.2 – Rattus norvegicus Kv1.2 channel; RatNav1.2II – Rattus norvegicus Type 1.2  sodium channel, homology domain II; RatNav1.4II – Rattus norvegicus Type 1.4 sodium channel,  homology domain II*). The highly conserved acidic residues in S2 and S3 are in open rectangles, while the characteristic positive arginine/lysine repeats (R1–R6) in the voltage sensor are shaded in black. *N.at-K*_*v*_*3.2 *has a histidine (H325) at position R1 (blue) and a glycine (G331) at position R3 (red). The characteristic potassium selective GYG sequence is highlighted in grey in the alignment of the canonical pore.

The flatworm channel *N.at-Kv3.2 *is a weak inward-rectifier that is open at hyperpolarized potentials, but exhibits mild rectification, limiting the efflux of potassium, at more depolarized potentials [17]. The amino acid sequence of *N.at-Kv3.2 *resembles other Shaker superfamily channels, in particular the Shaw (Kv3) family [17]. However, the voltage sensor in *N.at-Kv3.2 *differs from other Kv channels in that the first (R1) and third (R3) basic residue positions are not arginine or lysine, but histidine (H325) and glycine (G331) respectively (Figure 1). Despite the highly conserved canonical pore domain, *N.at-Kv3.2 *is permeable to a number of cations including potassium, caesium, lithium, guanidinium and, to a limited extent, barium and sodium. This channel's low ion selectivity raised the possibility that the current mediated by *N.at-Kv3.2 *might be passing through a permeability pathway other than the canonical selectivity filter, and the presence of two unusual amino acids in S4 suggested that this pathway could be a naturally occurring equivalent of the modified gating-pore that gives rise to the omega current in Drosophila Shaker channel mutants.

## Results and Discussion

### Ions are conducted through *N.at-K*_*v*_*3.2 *by a modified gating channel

To elucidate the contributions of the H325 and G331 residues to the alternate ion permeation pathway of *N.at-Kv3.2*, we created a series of mutants, based in part on the Shaker mutants created by Tombola and colleagues [12], that included a mutation to arginine, the probable ancestral residue, and constructed pair-wise combinations of all the mutations at H325 and G331 (Table 1).

**Table 1:** Summary table of the observed phenotypes and conductance properties of the N.at-Kv3.2 single and double S4 mutants

Channel	n	Phenotype	Delayed Rectifier Properties*
Wild-type	8	Inward rectifier	
H325A	5	Delayed rectifier	V_50 _= 51.9 ± 3.4 b = 21.8 ± 1.6
H325C	5	Delayed rectifier	V_50 _= 62.5 ± 5.7 b = 22.9 ± 2.1
H325S	4	Delayed rectifier	V_50 _= 60.6 ± 4.5 b = 19.2 ± 1.9
H325R	7	Delayed rectifier	V_50 _= 69.9 ± 8.8 b = 27.3 ± 2.7
H325E	18	Inward rectifier	
H325V	12	Inward rectifier	
G331P	6	Inward rectifier	
G331A	14	Inward rectifier	
G331R	7	Delayed rectifier	V_50 _= 73.8 ± 9.7 b = 23.8 ± 2.8
G331K	12	Delayed rectifier	V_50 _= 36.0 ± 1.0 b = 15.2 ± 0.8
H325A + G331P	8	Inward rectifier	
H325C + G331P	6	Inward rectifier	
H325S + G331P	6	Inward rectifier	
H325R + G331P	11	Inward rectifier	
H325E + G331P	6	Inward rectifier	
H325V + G331P	8	Inward rectifier	
H325A + G331A	9	Inward rectifier	
H325C + G331A	7	Inward rectifier	
H325S + G331A	7	Inward rectifier	
H325R + G331A	8	Inward rectifier	
H325E + G331A	8	Inward rectifier	
H325V + G331A	9	Inward rectifier	
H325A + G331R	8	Inward rectifier	
H325C + G331R	7	Inward rectifier	
H325S + G331R	n/a	No detectable current	
H325R + G331R	7	Inward rectifier	
H325E + G331R	10	Inward rectifier	
H325V + G331R	10	Inward rectifier	
H325A + G331K	12	Combined	V_50 _= 22.5 ± 1.8 b = 15.7 ± 1.5
H325C + G331K	7	Combined	V_50 _= 15.6 ± 3.4 b = 23.7 ± 2.8
H325S + G331K	6	Combined	V_50 _= 7.6 ± 0.4 b = 15.0 ± 0.4
H325R + G331K	6	Delayed rectifier	V_50 _= 25.6 ± 1.2 b = 16.5 ± 1.0
H325E + G331K	7	Combined	V_50 _= -27.5 ± 2.1 b = 23.3 ± 2.1
H325V + G331K	7	Combined	V_50 _= 12.1 ± 1.2 b = 10.2 ± 1.1

Channels were expressed in *X. laevis *oocytes and their electrophysiological properties were evaluated. V_50 _and Boltzmann slope factor were obtained by fitting to the delayed rectifier components of the current-voltage data.

**Figure 2:**
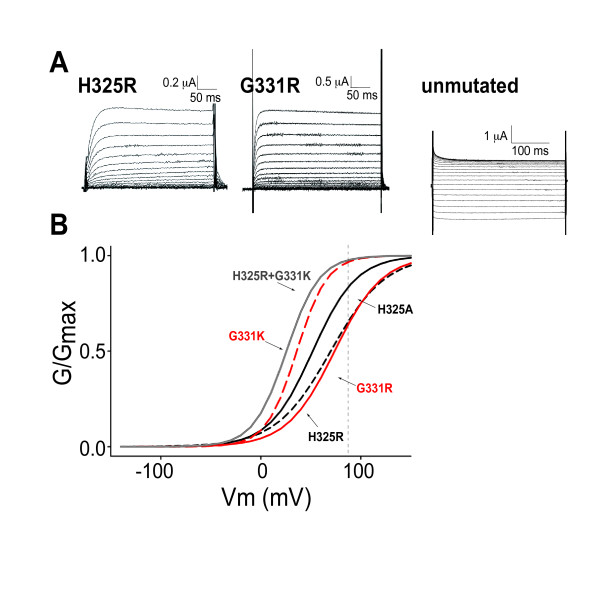
**Atypical residues at two positions in S4 of *N.at-K_v_3.2 *are responsible for the omega current**. A. Representative traces from single mutants H325R and G331R and the unmutated *N.at-K_v_3.2 *channel. Both mutants express delayed-rectifying phenotypes through 'rescue' of the canonical pore pathway, with outward current increasing monotonically with more positive voltages. The unmutated channel currents plateau and then decrease with more positive voltages. B. Fitted conductance-voltage relationships for the delayed-rectifier mutant channels. The individual data points are not shown, to avoid obscuring the fitted curves. The H325 single mutants are shown in black (H325A solid; H325R dashed), the G331 single mutants are shown in red (G331R solid; G331K dashed) and the double mutant H325R+G331K is shown in grey. The vertical dashed line indicates the upper limit (+90 mV) of stimulation voltages; the portions of the curves to the right are extrapolations.

**Figure 3:**
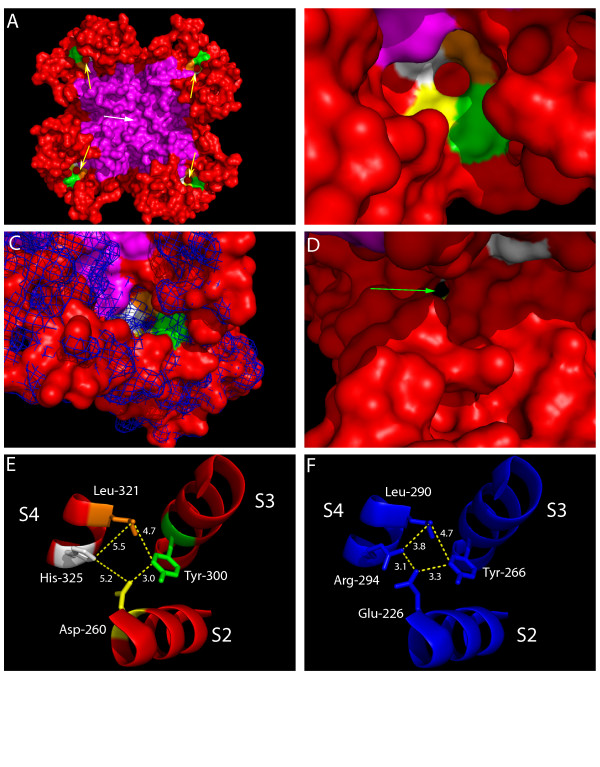
**Homology model of the *N.at-K*_*v*_*3.2 *channel on the closed state model of rat *K*_*v*_*1.2 *proposed by Pathak and colleagues**. Panel A. Extracellular view. The pore domains (S5-pore loop-S6) are magenta, the voltage sensor domains (S1–S4) are red and the four residues that bound the non-selective pore are white (His-325) orange (Leu-321) green (Tyr-300) and yellow (Asp-260). The central ion selective pore is indicated by the white arrow while the four proposed non-selective pores are indicated with yellow arrows. Panel B. Closer view of one of the non-selective pores. Portions of the intracellular part of the voltage sensing domain (red) are seen through the pore. Panel C. The surface of the aligned rat Kv1.2 closed state model is represented with a blue mesh, indicating that the gating pore is completely occluded in a typical delayed rectifier channel. Panel D. View of the nonselective pore (green arrow) from the cytoplasmic aspect of the channel protein. Gly-331 is the white surface on the upper right of the panel. Panels E and F. Inter-residue distances in the region of the gating pore in NatKv3.2 (panel E) and rat Kv1.2 (Panel F). It is the combination of a shorter side-chain in Asp-260 with a shorter and more compact His-325 that provides a pore that is sufficiently large to be permeable to hydrated ions (panel E), compared with an occluded pore created by Glu-226 and Arg-294 in delayed rectifier channels (panel F).

Four different single mutations of the histidine at position R1 (H325A/S/C/R) created a delayed-rectifier phenotype typical of Kv3 voltage-gated potassium channels (Figure 2A and Table 1). Two mutations of the glycine residue in S4 (G331R/K) also created delayed-rectifier channels. The fact that several mutations in the S4 helix caused a change from weak inward rectification to K_*v*_3 delayed rectification supports the idea that the wild-type *N.at-K*_*v*_*3.2 *conductance is mediated by four modified gating pores, rather than the single canonical permeability pore. This hypothesis is illustrated by a homology model of the *N.at-K*_*v*_*3.2 *sequence (Figure 3) on a recently developed model of the closed state of the rat *K*_*v*_*1.2 *channel [18]. His-325 is one of four residues that are positioned at a location homologous to the gating pore (Figure 3A, B, E). Compared to the rat *K*_*v*_*1.2 *structure, the smaller size of His-325 compared to Arg-294 contributes to a potential conduction pathway at the positions of the gating pores (Figure 3E, F).

Each of the mutations that convert *N.at-K*_*v*_*3.2 *to a delayed rectifier could contribute to occlusion of a gating pore conductance pathway by a different combination of several mechanisms, a) sterically blocking the pathway with a larger side chain (e.g. H325R), b) repelling cations by virtue of a positive charge on the side chain (e.g. H325R) and c) straightening a bend in the S4 helix caused by the presence of glycine at position 331 (e.g. G331K and G331R). Neither of the mutated sites is close enough to the canonical pore for any of these mechanisms to directly affect ion conduction through that pathway.

**Figure 4:**
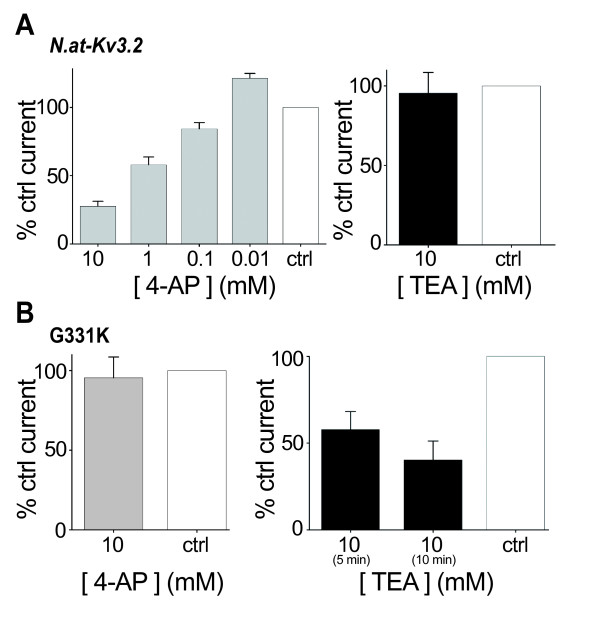
**Pharmacological characterization defines two permeation pathways in *N.at-K*_*v*_*3.2***. A. Treatment of the wild-type *N.at-Kv3.2 *with 4-aminopyridine (4-AP) blocked the inward rectifying omega current in a dose-dependent fashion, while treatment with 10 mM tetraethylammonium (TEA) failed to block this permeation pathway. Pharmacological block was calculated for each individual experiment as % control current, where the control was ND96 (n = 7). B. Treatment of the G331K delayed-rectifier mutant demonstrated TEA block (10 mM) of the canonical pore. This mutant channel was insensitive to 10 mM 4-AP (n = 6). The slow onset of the TEA block does not reflect slow intrinsic binding kinetics, but rather the time it takes the perfusate to come to equilibrium (See Taglialatela and colleagues [20] for a similar result with the rat drk1 channel). Error bars denote s.e.m.

Based on the amino acid sequence at the canonical pore's external vestibule (Figure 1) we expected that ion permeation through the canonical pathway would be blocked by TEA [19,20]. Conversely, permeation through an alternative ionic pathway should be unaffected by TEA. Figure 4A shows that the wild-type *N.at-Kv3.2 *was insensitive to TEA treatment up to a concentration of 10 mM. On the other hand the G331K mutant, which manifests delayed rectifier behaviour, was inhibited by 10 mM TEA, as would be predicted by the amino acid sequence of the canonical pore. However, the inhibition occurs in a time-dependent manner, suggesting that the substantial difference in amino acid sequence between the *N.at-K*_*v*_*3.2 *pore vestibule and the pore vestibules of other channels may be interfering with TEA binding. These results are consistent with the Shaker omega current mutants, where the omega current is insensitive to TEA while the canonical pore is blocked by application of the drug [12].

**Figure 5:**
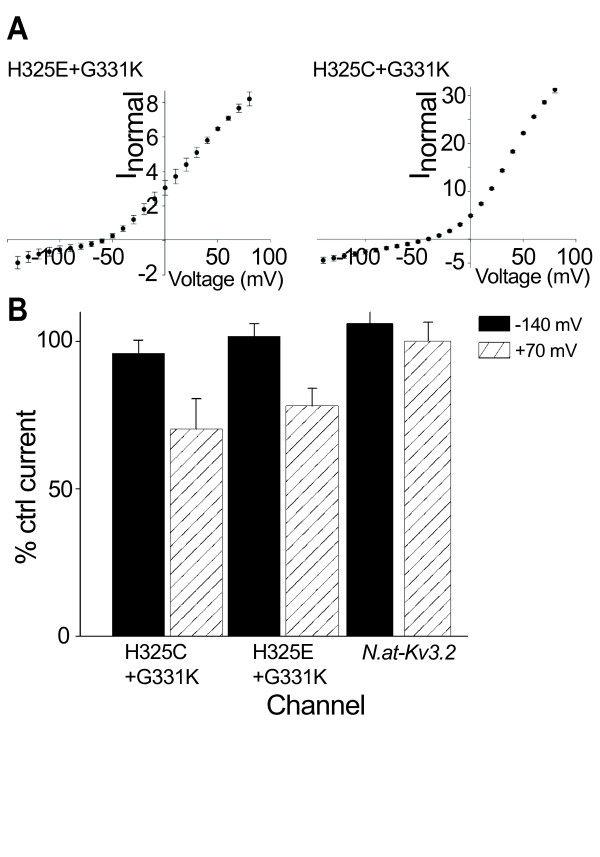
**The double H325E+G331K and H325C+G331K mutants manifest two permeation pathways within the same channel**. A) Current-voltage relationship for H325E+G331K and H235C+G331K double mutants display inward current in the voltage range where maximum inward rectification is observed in *N.at-K*_*v*_*3.2*. However, at more depolarizing voltages the current increases in a pattern typical for a voltage-activated delayed rectifier channel. B) Channels were treated with 10 mM TEA for 10 min. to block the delayed rectifier component of the dual phenotype channels. Pharmacological block was calculated for each individual experiment as % control current (where the control is ND96) at two voltages; -140 mV (filled bars) representing the omega current, and +70 mV (patterned bars) representing the delayed rectifier component. The delayed rectifier component (+70 mV current) of H325C+G331K and H325E+G331K mutants was blocked by 10 mM TEA while the wild-type was unaffected (n = 6, error bars denote s.e.m.). The current through the gating pore (-140 mV) remained unaffected in both mutants and the wild-type channel.

In contrast, the *N.at-Kv3.2 *wild-type channel was inhibited, in a dose-dependent manner, by 4-AP (10 μM to 10 mM; Figure 4A), while the G331K delayed rectifier was insensitive to 4-AP (Figure 4B). This indicates that the G331K mutant, which can be considered to be a back-mutation to a functional ancestral delayed-rectifier channel, passed potassium through the canonical pore, which was blocked by TEA but not by 4-AP, whereas, the wild-type channel passed current through a different pore, which was blocked by 4-AP but not TEA. We propose that in the *N.at-K*_*v*_*3.2 *channel the gating pores are binding sites for 4-AP, in contrast to most other delayed rectifier channels, in which 4-AP targets the canonical ion selective pore [21]. Although 4-AP has been shown to bind to the intracellular vestibule of the open canonical pore in the Shaker channel [21], in other channels it has been reported to bind to the closed state [22,23], and in one case to two different sites in a snail potassium channel [24]. The wild-type channel failed to manifest a TEA sensitive current even under large (+70 mV) depolarizations (Figure 5B).

These results indicate that the wild-type *N.at-Kv3.2 *channel utilizes the gating-pores as permeation pathways. In this interpretation the single mutations that convert the *N.at-Kv3.2 *channel to a delayed-rectifier both block the gating-pore, thus preventing an omega current, and either permitting the interaction between S4 and the canonical pore gate that is necessary for depolarization-dependent gating or, possibly, shifting the voltage sensitivity of gating leftward, into the physiological range, by establishing electrostatic interactions within the voltage-sensing domain that tend to differentially stabilize the open state relative to the closed state.

### The conductance-voltage curves of the delayed rectifying mutants are strongly right-shifted compared with other VKCs

Since the modified gating pore appears to have evolved as the ion-conducting pathway in *N.at-Kv3.2 *it would seem reasonable that permeation through the canonical pore would have been simultaneously selected against. The most depolarization sensitive of the single mutant delayed rectifier channels was G331K, with a half activation voltage (V_50_) of +36.0 ± 1.0 mV, compared with G331R, with a V_50 _of +73.8 ± 9.7 mV (Figure 2B, Table 1); V_50 _values for the other pure delayed rectifiers range from +51.9 to +69.9 mV. Thus, it appears that the evolutionary changes that have created the gating pore conductance pathway may have also shifted the voltage sensitivity of the voltage sensor domain into a range that is so positive relative to normal membrane potential that the canonical pore has become irrelevant to channel function under normal cellular conditions.

Conversion of the gating pore to an ion conducting pathway, and the consequent rightward shift of voltage-gated opening of the canonical pore, appears to be due to changes in both the packing of the amino acids in the voltage-sensing module of the channel and the charge content of the S4 helix. For example, both G331K and G331R mutations add a single positive charge to the voltage sensor, but the two have a difference in V_50 _value of ~37 mV. This means that the channel with the smaller added side-chain, lysine, is significantly more stable in the open state than the channel with the larger side-chain, arginine. The straightforward explanation for this result is that the sterically bulkier arginine sidechain requires more energy to pack it into the open state conformation, and thus has a more right-shifted V_50 _value. In addition to the unusual residues at position 325 and position 331, *N.at-Kv3.2 *also has unexpected acidic residues in the S2 and S3 helices. Residues 260 and 270 are both aspartate, compared with the almost universal presence of glutamate at the homologous positions in VKCs. Also, residue 293 is glutamate in *N.at-Kv3.2 *as compared to aspartate in the majority of other channels of this family (Figure 1). These acidic residues are central determinants of voltage sensitivity in Shaker; they differentially form salt bridges with the basic residues of S4 in the open and closed states [25-28]. Thus, it appears likely the *N.at-Kv3.2 *channel has undergone a number of evolutionary sequence changes that have created ion-conducting pores from the gating pore and moved the sensitivity of the voltage sensor into a range of voltages that are not likely to occur in most electrically excitable cells.

### Some double mutations of *N.at-Kv3.2 *manifest dual inward rectifier and delayed rectifier properties

The electrophysiological properties of the single R1 mutants in *N.at-Kv3.2 *are notably different from the *Drosophila Shaker *gating-pore mutants, in which ionic access to the gating-pore is permitted by R1S/C/V/A mutants while the canonical permeability pore also opens at supra-threshold potentials [15]. To further investigate the differences between the synthetically created omega current in *Shaker *and the naturally occurring omega current in *N.at-K*_*v*_*3.2 *we created pair-wise combinations of the single mutations. Of the 24 pairwise mutants, 17 were weak inward rectifiers, one did not express, five had a combination of inward and delayed rectification (See Figure 5A for examples) and one was a pure delayed rectifier.

Interestingly, the only double mutant that produced a purely delayed-rectifier in *N.at-K*_*v*_*3.2 *was H325R+G331K. The combination of basic residues at R1 and R3 in the voltage sensor shifted the voltage sensitivity of the resulting delayed rectifier leftward by 10 mV compared to the G331K mutation alone (V_50 _= 25.6 ± 1.2 mV; Figure 2B). In our interpretation, the addition of arginine at R1 and lysine at R3 re-establishes ancestral salt-bridge interactions with acidic residues in S2 and S3 (see above) that together stabilize the open gating conformation of the channel and at the same time influence localized protein packing so as to block the gating pore pathway, thereby preventing the influx of omega current. Since arginine in the R3 position of the H325R+G331R double mutant produced an inward rectifying channel, and the G331R single mutation produced a delayed rectifier with an extremely positive V_50_, it is apparent that the larger arginine side-chain causes steric crowding within the voltage-sensing domain. In this model crowding could 1) selectively destabilize the open state relative to the closed state, thus requiring a very positive transmembrane potential to provide sufficient energy for transition to the open state and 2) contribute to a conformation that has a sufficiently large opening in the gating pore to be non-selective for cations.

### Double mutants with combined inward rectification and delayed rectification manifest ion permeation through two pores, with different voltage dependencies and drug sensitivities

G331K-containing double mutants (H325A/C/S/V/E) displayed a combination of the two permeation pathways (Table 1). H325E+G331K and H325C+G331K inward non-selective cation current measured at -140 mV was unaffected by application of 10 mM TEA, while the delayed rectifier currents measured at +70 mV were suppressed by ~30% in the presence of the drug (Figure 5B). This duality in function is observed in *Drosophila Shaker*, Na_V_1.4 and Na_V_1.2 mutant channels, where a small omega current is observed at hyperpolarized potentials, while activation of the canonical pore at depolarized potentials produces a large alpha current [12,13,16].

The delayed-rectifier component of the G331K-containing double mutants had left-shifted V_50 _values compared to the G331K mutant alone. The H325A+G331K mutant had a V_50 _of 22.5 ± 1.8 mV, while the H325C+G331K, H325V+G331K, H325S+G331K and H325E+G331K mutants were shifted to the left by an additional 7, 10, 15 and 50 mV respectively compared to H325A+G331K (Table 1). The phenotypes of these channels were the most similar to the *Shaker *gating-pore mutants where R1A/C/S/V/E permits current flow through the gating pore at hyperpolarized potentials, while the canonical pore opens at depolarized potentials [12]. However, unlike the *Shaker *mutants, where modification at the R1 position has limited effect on voltage-sensitivity, the localized protein packing differences of the *N.at-Kv3.2 *G331K-containing double mutants had great impact on the voltage-sensing behaviour of the channel, suggesting that the wild-type *N.at-Kv3.2 *channel has evolved away from using the canonical pore. It is significant that all other combinations of double mutants produced only inwardly rectifying channels. These include combinations of mutations that singly produced delayed-rectifier channels (e.g. H325A+G331R, Table 1), indicating that most single mutations were not simple back mutations to the delayed rectifier phenotype. The fact that no single mutation in *N.at-K*_*v*_*3.2 *produced a delayed rectifier with a V_50 _that would function in a physiological range of membrane potential suggests that this channel has undergone several mutational changes since it diverged from the ancestral delayed rectifier. Many mutations that produced a delayed rectifier individually, in combination, produced an inward rectifier with properties very similar to the wild-type channel. This suggests that *N.at-K*_*v*_*3.2 *has undergone extensive evolutionary change that has selected for robust ion permeation through the gating pore-derived permeability pathway.

As in other VKCs, the voltage sensor in *N.at-Kv3.2 *has a number of basic residues that presumably move in response to changes in transmembrane voltage. As shown by Tombola and colleagues [12], as the voltage sensor moves in response to membrane depolarizations, the current through the gating pore diminishes. In our model of the weak inward-rectifier mutants, we propose that voltage-dependent movements of the voltage sensor that are insufficient to open the canonical, potassium-selective pore are able to re-conform the gating-pore pathway so as to restrict ion permeation through the gating-pore without blocking it, resulting in weak rectification.

### Mutations that engender only weak inward rectification cause quantitative changes in ion selectivity

**Figure 6:**
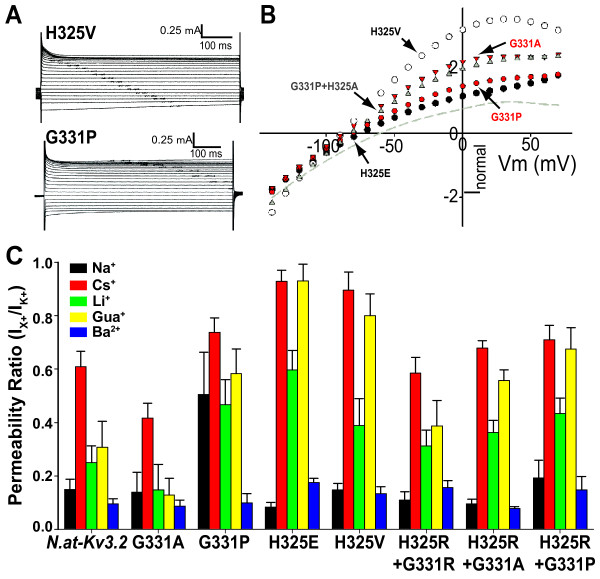
**Unique residues in S4 affect accessibility and the shape of the gating-pore pathway.**  A. Representative traces from single mutants H325V and G331P illustrating the persistence of inward  rectification and use of the gating-pore pathway. B. Current-voltage relationships for the inwardly rectifying single mutant channels. H325V (open circles) and H325E (black circles) have similar inward currents. H325V is a much weaker rectifier. The G331 single mutants are shown in red (G331P circles; G331A triangles) and the double mutant H325A+G331P is shown in grey triangles. The wild-type current-voltage relationship from 1B was renormalized with this dataset and is shown as a grey dashed line for reference. Error-bars have been removed for clarity. C. Permeation profiles for selected inward-rectifier channels. Permeability is shown as the fraction of the permeability of the channel to K^+^. n=4 for wild type, n=6 got G331A, n=6 for G331P, n=5 for H325E, n=7 for H325V, n=7 for H325R-G331R, n=6 for H325R-G331A, and n=6 for H325R-G331P. Error bars represent s.e.m.

The majority of double mutant *N.at-Kv3.2 *channels were weak inward rectifiers (Table 1), although there is substantial variation in the amount of inward current relative to outward current (Figure 6A, B). *N.at-Kv3.2 *is capable of passing a number of monovalent and divalent cations, including caesium and barium (Figure 6C), which block the canonical conductance pore. Guanidinium ions also pass through the alternative pathway in this channel, suggesting a large aqueous canal through the gating pore. Mutation of the histidine residue at position R1 changed the accessibility of the gating-pore to extracellular ions. The addition of an acidic residue at the entrance to the gating-pore (H325E) yielded an inward-rectifier with the least cation selectivity of all mutant channels, with both caesium and guanadinium having approximately 90% of the permeability of potassium at hyperpolarized potentials (Figure 6C). We hypothesize that side-chain repulsion between the negatively charged carboxyl groups of H325E and D260 (the first acidic residue in S2) opens the aperture at the gating-pore and attracts cations.

The shape of the gating-pore pathway was drastically changed by the replacement of G331 with a proline (which creates a kink with more limited flexibility in the helix) in the S4 helix. In G331P, all monovalent cation species were highly permeant relative to potassium, including Na^+^. Conversely, the hypothetically straight helix of the G331A mutant provided increased potassium selectivity, with an average permeability that was ~10 times greater for potassium than for the other cations, with the exception of caesium (Figure 6C). The selectivity of the gating pore pathway is related to ionic size and chemical properties as well as localized protein packing and side-chain interactions

When the conformation of the voltage sensor helix of the H325R mutant is constrained by the replacement of G331 with alanine, arginine, or proline, the permeability profiles of the gating-pore pathway were all qualitatively similar to each other (Figure 6C). The three S4 double mutants, H325R+G331P, H325R+G331A and H325R+G331R all had high potassium permeability at hyperpolarized potentials and the relatively high permeability for other cations that is characteristic of the gating pore pathway. Thus, it appears that the pair-wise mutations within the S4 voltage sensor disrupt the secondary structure of the voltage-sensing domain that blocks the alternative permeation pathway present in the H325R mutation.

## Conclusion

The inwardly rectifying 6TM K_*v *_channel, *N.at-K*_*v*_*3.2 *has evolved to utilize an ion permeation pathway other than the canonical pore, which is also present. We provide evidence that the alternate pathway is derived from the gating pore.

We show that 4-AP does not block K^+ ^current in the delayed rectifier mutants that are sensitive to TEA (Figure 4), and conversely, that TEA does not block K^+ ^current in the wild-type, weakly inward rectifying channel (Figure 4). We show that in channels that manifest a combination of the two types of permeability, only the outward current, at +70 mV, is affected by TEA, not the inward current, at -140 mV. Thus, it is clear that we are looking at two distinct ionic conduction pathways in this study, and that 4-AP is blocking the non-canonical pathway. The alternative interpretation, that the various mutations are acting by changing the canonical pore, would require fully reciprocal switching between 4-AP sensitivity and TEA sensitivity of the channel that is also correlated with an unprecedented loss of ion selectivity by the highly conserved canonical K^+ ^channel pore.

This unique permeation mechanism has evolved through changes to the basic residue content of the S4 helix, thus modifying the electrostatic interactions between the S4 voltage sensor and surrounding helices. The proteinaceous septum formed by the S1, S2, S3 and S4 helices, at the interface of the extracellular and intracellular aqueous vestibules of the gating-pore, is not present in *N.at-K*_*v*_*3.2*. We have demonstrated that the accessibility, shape and function of the gating-pore can be modified, and that the selectivity of the gating-pore pathway is related to ionic size and chemical properties of the permeant ion as well as localized protein packing and side chain interactions.

These results raise a novel possibility for functional evolution of ion channels. The omega current pathway has evolved in *N.at-K*_*v*_*3.2 *to produce a non-selective cation channel that is open at hyperpolarizing potentials. This is an example of a distinctive molecular functionality arising, not by quantitative variation of an existing molecular property, but rather by creation of a new molecular property. It may be that other hyperpolarization-activated, non-selective cation channels have evolved in other organisms by similar changes in one of the more typical voltage-activated ion channels.

## Methods

### Expression plasmids

The full-length *N.at-Kv3.2 *channel in pXT7 was used as a template for overlapping PCR site-directed mutagenesis [29] at amino acids 325 and 331. Double mutants were generated by ligating small fragments containing the various mutations at position 325 into plasmids containing the different mutants at position 331, using convenient restriction sites. All mutant plasmids were fully sequenced to confirm the desired mutation and the absence of PCR induced mutations.

Plasmids were linearized with XbaI and gel purified. Capped mRNAs were prepared by in vitro transcription using an mMessage mMachine (Ambion) T7 polymerase kit, and stored at -80°C.

### Oocyte preparation

An ovary removed from a 2 year old *Xenopus laevis *female was manually separated into half-centimeter clumps, rinsed three times in MBM (in mM: NaCl 88, KCl 1, Ca(NO3)2 0.33, CaCl2 0.41, MgSO4 0.82, NaHCO3 2.4, HEPES 10, sodium pyruvate 2.5, supplemented with penicillin G 0.1 g/L and gentamycin sulfate 0.05 g/L (pH 7.5 with Tris Base) [30]), incubated on a rotating shaker for 2 h in 2 mg/ml collagenase 1A (Sigma) in MBM at room temperature, rinsed in MBM and incubated, ~200 eggs/glass scintillation vial, at 17°C overnight in fresh MBM. Eggs were de-folliculated by immersion in a hypo-osmotic phosphate buffer (in mM: K2PO4 100 (pH 6.5 with HCl)) for 1 h and were left for 2 h in fresh MBM to recover prior to manipulation.

Mature stage V-VII oocytes were injected with 48.6 nl mRNA (200–600 ng/nl) and incubated in SOS+ (in mM: NaCl 96, KCl 2, CaCl2 1.8, MgCl2 1, HEPES 5, sodium pyruvate 2.5 (adjusted to pH 7.4), supplemented with 3% horse serum (Sigma) and gentamycin sulfate 0.01 g/ml) at 17°C.

### Two-electrode voltage clamp electrophysiology

Electrophysiological measurements were performed 1 day after injection for all channels except G331R, G331K, H325A, H325S, H325C, and H325R and G331K+H325R. These channels required 2 days of incubation to obtain measurable levels of current. Oocytes were impaled with glass microelectrodes fabricated from borosilicate glass, filled with 3 M KCl and having a resistance of 0.5 to 1 MΩ. Experiments were driven by a GeneClamp 500B amplifier (Axon Div., Molecular Devices) controlled by pClamp 9.0 software (Axon Div., Molecular Devices). Data were acquired through a 1322A Analogue/Digital converter and analyzed using Clampfit 9.0 (Axon Div., Molecular Devices).

Experiments were performed in ND96 (in mM: NaCl 96, KCl 2, CaCl2 1.8, MgCl2 1, HEPES 5, pH 7.4). Recordings on inward rectifier channels and combined delayed rectifier/inward rectifier channels measurements were performed without leak subtraction. Data for the delayed rectifier channels was obtained with leak subtraction (P/N = 4).

Steady-state currents for inward rectifier and delayed rectifier/inward rectifier channels were elicited by holding the oocyte at -90 mV for 50 ms, followed by a series of 400 ms depolarizations from -140 mV to +90 mV in 10 mV steps, followed by a return to a -90 mV holding potential.

Steady-state currents for delayed rectifier channels were elicited by holding the oocyte at -90 mV for 50 ms, followed by a series of 200 ms depolarizations from -140 mV to +90 mV in 10 mV steps followed, by a return to a -90 mV holding potential.

Current/voltage measurements were taken 10 ms before the capacitative transient at the end of the activation step. Data from individual experiments were normalized to an arbitrarily chosen single experiment by multiplying the individual current readings by the ratio of the sums of the currents measured from -140 mV to -90 mV.

To fit to the Boltzmann equation for channels that manifested a combination of inward rectifier and delayed rectifier properties, the inward component of the currents for the G331K double mutants was ignored. Steady state tail currents for traces at or near the reversal potential and subsequent traces through the depolarized voltage range were obtained, normalized and plotted against the voltage step that elicited the current. These conductance voltage plots were then fitted to the Boltzmann equation, with a y offset to accommodate the elevated conductance near the reversal potential, to obtain values for V_50 _and slope factor.

### Ion permeability

Perfusion experiments were performed to test ion permeability. In these ion substitution experiments the bath contained a 100 mM solution of a single cationic salt, guanidinium chloride (GuCl), LiCl, CsCl, NaCl, KCl, or BaCl2, and, in mM; HEPES 10; EDTA 1; pH 7.6. The relative permeability of each ion relative to the permeability for K^+ ^was calculated from the reversal potentials measured for the respective ions using the equation:

$$\frac{P_{X^{+}}}{P_{K^{+}}}=\exp(\frac{zF}{RT}(E_{rev,X^{+}}-E_{rev,K^{+}}))$$

for monovalent cations [31-33] and the equation:

$$\frac{P_{X^{++}}}{P_{K^{+}}}=\frac{1}{4}\times \exp\{\frac{(E_{rev,X^{++}}-E_{rev,K^{+}})F}{RT}\}\times \exp\{\frac{E_{rev,X^{++}}F}{RT}+1\}$$

for divalent cations [34]. We found that incubation with Ba^++ ^caused large changes in permeability to other ions, so Ba^++ ^was always the last ion tested on each oocyte.

### Pharmacology

Pharmacological experiments for tetraethylammonium (TEA) and for 4-aminopyridine (4-AP) were performed by creating a 100 mM stock of TEA or 4-AP in ND96 saline, and creating serial dilutions for the concentrations specified. These solutions were then perfused into the bath and left to incubate for the time specified.

### Molecular modelling

The alignment of the *N.at-K*_*v*_*3.2 *amino acid sequence and the rat *K*_*v*_*1.2 *sequence was extracted from a multiple alignment of 790 voltage-gated potassium channel sequences. The cytoplasmic T1 domain sequence was truncated and the resulting alignment was used to create a homology model of *N.at-K*_*v*_*3.2 *on the closed state model of the rat *K*_*v*_*1.2 *channel proposed by Pathak and colleagues [18] using SWISS MODEL [35].

## Authors' contributions

TLK conceived and executed the experiments, performed the data analysis and wrote the paper. ANS contributed to experimental design and wrote the paper. WJG contributed to experimental design, participated in data analysis, performed the homology modelling, and wrote the paper. All authors have read and approve the final manuscript.
